# Toll-Like Receptor 4 (TLR4) and Typhoid Fever in Vietnam

**DOI:** 10.1371/journal.pone.0004800

**Published:** 2009-03-11

**Authors:** Nguyen Thi Hue, Mai Ngoc Lanh, Le Thi Phuong, Ha Vinh, Nguyen Tran Chinh, Tran Tinh Hien, Nguyen T. Hieu, Jeremy J. Farrar, Sarah J. Dunstan

**Affiliations:** 1 Oxford University Clinical Research Unit, Hospital for Tropical Diseases, Ho Chi Minh City, Vietnam; 2 Hospital for Tropical Diseases, Ho Chi Minh City, Vietnam; 3 Dong Thap Provincial Hospital, Dong Thap, Vietnam; 4 Centre for Tropical Medicine, Nuffield Department of Clinical Medicine, Oxford University, Oxford, United Kingdom; 5 Hung Vuong Hospital, Ho Chi Minh City, Vietnam; University of Toronto, Canada

## Abstract

Understanding the host genetic susceptibility to typhoid fever may provide a better understanding of pathogenesis and help in the development of new therapeutics and vaccines. Here we determine the genetic variation within the human *TLR4* gene encoding the principal receptor for bacterial endotoxin recognition in typhoid fever patients. It is possible that genetic variants of *TLR4* could detrimentally affect the innate immune response against *S. typhi* infection. Mutation detection and genotyping of *TLR4* was performed on DNA from 414 Vietnamese typhoid fever patients and 372 population controls. dHPLC detected a total of 10 polymorphisms within the upstream and exonic regions of *TLR4*, of which 7 are novel. Two SNPs, T4025A and C4215G, were more frequent in typhoid cases than in controls however due to their low allele frequencies they showed borderline significance (T4025A: OR 1.9, 95%CI 0.9–4.3, *P* 0.07 and C4215G: OR 6.7, 95%CI 0.8–307, *P* 0.04). Six missense mutations were identified, with 5/6 positioned in the ectoplasmic domain. Four missense mutations and one promoter SNP (A-271G) were only present in typhoid cases, albeit at low allele frequencies. Here we determined the extent of genetic variation within *TLR4* in a Vietnamese population and suggest that *TLR4* may be involved in defense against typhoid fever in this population.

## Introduction

*Salmonella enterica* serovar Typhi (*S.* Typhi), is a Gram negative bacterium that can cause typhoid fever [1]. Worldwide typhoid fever is a serious public health problem, with an estimated 22 million cases, resulting in 200,000 deaths [2]. The burden of disease lies mainly in developing countries where the provision of sanitary conditions can be inadequate. It has been reported that multi-drug resistant strains of *S.* Typhi are emerging, posing a real threat to the current antimicrobial treatment of typhoid [1]. To be in a position to develop novel therapeutic agents or new vaccines for typhoid it is imperative that the immunological mechanisms of disease protection are thoroughly understood. The availability of the human genome sequence and advances in molecular technologies allows the study of protective disease mechanisms using a genetic approach.

Genetic susceptibility to typhoid fever has until recently been predominately studied in a mouse model of infection. Initially it was observed that certain inbred mouse strains were innately susceptible to *Salmonella enterica* serovar Typhimurium (*S.* Typhimurium), the bacterium that causes a systemic disease in mice that mimics human typhoid [3]. One such inbred strain, C3H/HeJ has a defective response to bacterial endotoxin [4], and macrophages from these mice fail to induce inflammatory cytokines after exposure to lipopolysaccharide (LPS). Poltorak *et al* (1998) [5] identified that the defective LPS signaling in these mice was due to a mutation in the *Tlr4* gene and Hoshino *et al* (1999) [6] produced further evidence by generating *Tlr4*-deficient mice that were phenotypically similar to C3H/HeJ mice. Infecting *Tlr4*-deficient mice intraperitoneally and orally with *S.* Typhimurium confirmed that Tlr4 contributes to murine host defense against *Salmonella* [7], [8].

Human TLR4 is recognized as the principal receptor for bacterial endotoxin recognition. It is one of 10 TLRs that upon stimulation activates the transcription factor nuclear factor κ-B (NFκ-B) and a signaling cascade that leads to the increased expression of immune and pro-inflammatory genes [9]. TLRs thereby play an essential role in innate and adaptive immunity [10] with TLR4 contributing to the early detection and immune response to Gram-negative infection.

Co-segregating missense mutations (Asp299Gly and Thr399Ile), that affect the extracellular domain of TLR4, were originally reported to be associated with a blunted response to inhaled LPS in humans [11]. Controversially, a more recent study demonstrated that monocytes from Asp299Gly heterozygotes exhibit no deficit in LPS recognition [12]. A number of studies however have shown that *TLR4* Asp299Gly is associated with a variety of infectious diseases, including septic shock [13], RSV [14], Legionnaires' disease [15] and malaria [16], but is not associated with others, such as tuberculosis [17] or meningococcal disease [18] (see table 1). Although *TLR4* Asp299Gly was not associated with meningococcal disease, it has been subsequently reported that other rare *TLR4* mutations may contribute to meningococcal susceptibility [19]. Prior to this finding, Smirnova *et al* (2001) [20] had investigated the sequence variation throughout *TLR4* to find an unusual excess of low frequency amino acid variants.

**Table 1:** Summary of TLR4 polymorphisms associated with infectious diseases.

Association study	SNP	Sample size (case/control)	Allele frequency (%) in cases vs controls	*P* value	Reference
Septic shock	Asp299Gly (rs4986790)	91/73	5.5 vs 0	0.05	[13]
Systemic inflammatory response syndrome	Asp299Gly	94/0	Increased mortality in patient carrying minor allele (19.0 vs 5.0)	0.076	[45]
Server sepsis following burn injury	Asp299Gly	159/0	SNP increased risk of developing severe sepsis	0.01	[46]
Respiratory syncytial virus infection	Asp299Gly	99/82	20.2 vs 4.9	0.003	[14]
	Thr399Ile (rs4986791)	99/90	20.2 vs 5.6	0.004	
Legionnaires' disease	Asp299Gly	108/508	4.9 vs 12.9	0.025	[15]
	Thr399Ile				
Meningococcal disease	Asp299Gly	252/251	20.2 vs 20.3	0.9	[47]
	Thr399Ile			NS	
Meningococcal disease	Asp299Gly	1047/879	5.9 vs 6.5	NS	[18]
Malaria	Asp299Gly	290/290/290	24.1 vs 17.6	<0.05	[16]
	Thr399Ile		6.2 vs 2.4	0.02	

We have previously investigated TLR5 deficiency in patients with typhoid fever [21]. The frequency of TLR5^392STOP^, which functions as a dominant negative and severely impairs signaling, was not significantly associated with typhoid fever. Despite *in vitro* and murine studies detailing the recognition of *Salmonella* flagellin by TLR5, this pattern recognition molecule may not play an important role in TLR-stimulated innate immune responses to human infection with *S.* Typhi. Initiation of these responses may rely on other TLRs recognizing different bacterial ligands. Numerous studies investigating the role of *Tlr4* in the mouse model of *Salmonella* infection, and cellular studies identifying how TLR4 recognises *Salmonella* LPS have been reported, however there have been no human studies investigating the contribution of *TLR4* to the genetic susceptibility to typhoid fever. Here we report the detection and genotyping of mutations within the *TLR4* gene in typhoid fever patients and controls in the Vietnamese population. We postulate that genetic variation within *TLR4* may effect recognition of *S. typhi* LPS, altering activation of innate immunity and hence detrimentally affecting the first line of defense against this pathogen.

## Materials and Methods

### Study populations

Genomic DNA from patients with typhoid fever was collected as part of larger epidemiologic or treatment studies. These studies were either performed at the Hospital for Tropical Diseases in Ho Chi Minh City, Dong Thap Provincial Hospital in Dong Thap province or Dong Nai Paediatric Center in Dong Nai province. Venous blood was collected from 414 patients with blood culture positive typhoid fever admitted to one of the three hospitals. The samples and studies have been described previously [22]. The 372 control DNA samples used in this study were extracted from umbilical cord blood samples from babies born at Hung Vuong Hospital in Ho Chi Minh City during 2003.

All case patients and control subjects were unrelated and were of the Vietnamese Kinh ethnicity. There were no significant gender differences between the case and control groups (male cases = 48.9%, male controls = 51.1%, female cases = 54.3%, female controls = 45.7%; χ^2^ = 2.314, *P* = 0.128).

### Ethics Statement

This study was conducted according to the principles expressed in the Declaration of Helsinki. Written informed consent was obtained from the individuals admitted into the study. For cord blood samples written parental consent was obtained. Ethical approval was obtained from the Ethical and Scientific Committee of the Hospital for Tropical Diseases, the Dong Thap Hospital and the Health services of Dong Thap Province and the institutional review board of Dong Nai Paediatric Center. Ethical approval was also granted from the Oxford Tropical Research Ethics Committee (OXTREC) of Oxford University, UK.

### DNA extraction and quantification

Genomic DNA from typhoid patients and cord blood was extracted from venous blood using either the QIAamp DNA blood midi kit (Qiagen) or the Nucleon BACC1 extraction kit (Nucleon Biosciences). DNA concentration was determined by UV absorbance at OD 260 nm using an Eppendorf Biophotometer (Eppendorf).

### Genotyping TLR4 Asp299Gly by allele-specific PCR

*TLR4* A896G (Asp299Gly) was genotyped by allele-specific PCR using conditions previously described [21]. The A allele-specific primer 5′-AGACTACTACCTCGATG**A** and the G allele-specific specific primer 5′-AGACTACTACCTCGATG**G** were used together with the consensus primer 5′-GCATTCCCACCTTTGTTGG to amplify an allele-specific fragment of 218 bp.

### Mutation Detection by dHPLC

**Figure 1:**
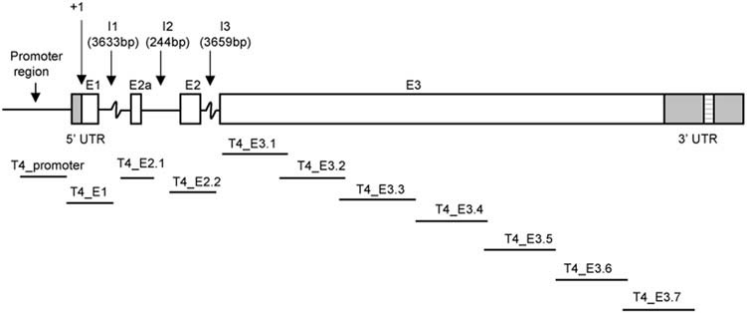
*TLR4* gene structure and fragment design for dHPLC. E1, E2, E3 denotes exons 1, 2, and 3. E2a denotes an alternative exon 2. I1, I2, I3 denotes intronic sequences. 5′UTR and 3′UTR represent the untranslated regions. The lines underneath the gene structure show the approximate positions of the 11 fragments designed for mutation detection by dHPLC. The names of each fragment are above the lines.

Primers and amplicons were designed to detect mutations in TLR4 by dHPLC according to the recommendations described by the manufacturer (Wave® DNA Fragment Analysis System, Transgenomic, USA) (table 2). The 5′ upstream regulatory region and 3 exons of the TLR4 gene were divided into 11 fragments (figure 1). The length of the fragments ranged from 338 bp to 506 bp, and the length of primers ranged from 18 bp to 25 bp, with each primer having a melting temperature (Tm) of approximately 56°C (figure 1, table 2). All primers were designed using Primer Prediction and Analysis programs found at http://www.hgmp.mrc.ac.uk/GenomeWeb/nuc-primer.html. To design the final primers for fragment generation, the melting temperature of the fragment had to be predicted using the WAVEMARKER 2000XL software (Transgenomic, USA). Based on the fragment's predicted melting temperature GC rich clamps were added to 4 primers to improve the melting temperature profile of the corresponding amplicon (table 2).

**Table 2:** Primers used to generate amplicons for mutation detection in *TLR4* by dHPLC.

Fragment	Size	Primers	Tm	GC%	2Td
T4_ promoter	421 bp	F 5′-AAAATGAATGTCTGTTGTTTAAGC-3′	56.72	29.17	57.5
		R 5′-GTGTCTTCTCTTCCTCGAGC- 3′	56.73	55	
T4_ E1	338 bp	F 5′-AAGTCCAGAATGCTAAGGTTG-3′	56.14	42.86	62
		R 5′-GCAGAAGTGAGGGAAAGTTC-3′	56.54	50	
T4_ E2.1	338 bp	F 5′-AGCAAGCACGATATTGGATA-3′	55.94	40	56
		R 5′-GTCCATCCTTCCATCCATAT-3′	56.13	45	
T4_ E2.2	432 bp	F 5′-AGCATGAATTGAGTGAATGG-3′	56.11	40	58
		R 5′-CAAACCAAGCTTTCCAGTC-3′	55.87	47.37	
T4_ E3.1	438 bp+10 bp	F 5′-ATGAAGAGCTGGATGACTAGG-3′	56.08	47.62	57
		R 5′-1 **CCCCCGCCCG**AGGGGCATTTGATGTAGAAC-3′	56.59	45	
T4_ E3.2	502 bp	F 5′-TGAGTATTTTTCTAATCTGACCAATC-3′	57.17	30.77	55.5&56
		R 5′-TAAATGTTGCCATCCGAA-3′	55.94	38.89	
T4_ E3.3	484 bp+10 bp	F 5′-**GCCCCCGCCG**GCATACTTAGACTACTACCTCGATG-3′	55.77	44	56
		R 5′-GGTAAATGAGGTTTCTGAGTGATA-3′	56.56	37.5	
T4_ E3.4	477 bp+10 bp	F 5′-**GCCCCCGCCG**AACCAGCCTAAAGTATTTAGATCTG-3′	56.62	36	58
		R 5′-ATTATGTGATTGAGACTGTAATCAAG-3′	55.84	30.77	
T4_ E3.5	462 bp	F 5′-CACTCTCCAGTCTTCAGGTACTAA-3′	56.89	45.83	56.5&57
		R 5′-TTTCACCTCTACCATACTTTATGC-3′	56.7	37.5	
T4_ E3.6	400 bp+10 bp	F 5′-**GCCCCCGCCGA**TTGGTGTGTCGGTCCTC-3′	56.65	55.56	59.5
		R 5′-TGATACCAGCACGACTGC-3′	56.67	56.67	
T4_ E3.7	506 bp	F 5′-GTTTCCATAAAAGCCGAAAG-3′	56.57	40	59&59.5
		R 5′-GGAAGCTCCTTGAGATTAGC-3′	55.85	50	

1 nucleotides in bold represent GC clamps added to the primer to improve the melting profile of the generated amplicon for mutation detection.

2 the temperatures used in dHPLC for each fragment to accurately identify sequence changes in TLR4.

PCR for each fragment was performed by using a mix of 2.5 µl of PCR buffer (10×), 1.5 µl of MgCl_2_ (25 mM), 0.25 µl of dNTPs (25 mM) each, 0.15 µl of each primer (20 mM), 0.3 µl of Taq polymerase (Taq Gold∶ Pfu = 2∶1), 6.5 µl DNA 15 ng/µl and 6.75 ul H_2_O. A touch-down PCR was performed using a temperature cycle of 95°C for 12 minutes, then 10 cycles of 95°C for 30 seconds, 64°C for 1 minute, decrease 0.5°C for each cycle, then 20 cycles of 95°C for 30 seconds, 59°C for 1 minute, 72°C for 1 minute, then 72°C for 5 minutes. The PCR products were hybridised for dHPLC by denaturation and slow annealing (95°C for 1 minute, decreasing 2°C for each cycle, for 35 cycles).

After hybridization the PCR products could be analysed by dHPLC. Performing a melt curve of 5 temperatures around the predicted temperature [predicted using WAVEMARKER 2000 software (Transgenomic, USA)], on pools of 5 samples, for all 11 fragments, provided the actual temperature used to separate heteroduplexes from homoduplexes. Table 2 shows the temperatures used in dHPLC for each fragment to accurately identify sequence changes in TLR4. Once the actual melting temperature for each fragment was determined, each fragment of every sample was analysed by dHPLC individually.

### DNA sequencing

DNA sequencing was performed by capillary electrophoresis using a CEQ8000 according to the manufacturer instructions (Beckman Coulter, Singapore).

### Statistical Analysis

Pearson's χ^2^ test was used to test associations between disease phenotypes and allele or genotype frequencies using STAT/SE 8.0 (Stata Corporation, Texas, USA). The Fisher's exact test was used when an expected value in the contingency table was <5. *P*<0.05 was considered significant.

## Results

### Genotyping TLR4 Asp299Gly in Typhoid patients and controls

We genotyped the previously reported *TLR4* Asp299Gly mutation in typhoid cases and controls. The frequency of Asp299Gly is ∼10% in Caucasians [23] and as high as 21.5% in Ghanian Africans [16] but has not previously been determined in Asian populations. Asp299Gly was completely absent in a subset of 372 typhoid cases and 372 controls (data not shown) indicating a frequency of below 1% in the Vietnamese population. As this polymorphism was not common in the Vietnamese we chose to identify which *TLR4* polymorphisms exist in the Vietnamese Kinh population (the largest ethnic group in Viet Nam).

### Detecting TLR4 mutations

Initially mutation detection by dHPLC was performed for each individual typhoid case (N = 93) and control sample (N = 93) for each of the 11 fragments spanning TLR4. If a dHPLC trace different to the wild-type dHPLC trace was identified, then mutation detection was continued for that fragment individually in the remaining 279 cases and 279 controls. If no different dHPLC pattern was detected then mutation detection for that individual fragment was discontinued. To confirm that a different dHPLC trace actually represented a polymorphism, all dHPLC patterns identified were grouped and the DNA of 1–3 samples from each pattern group was sequenced.

**Figure 2:**
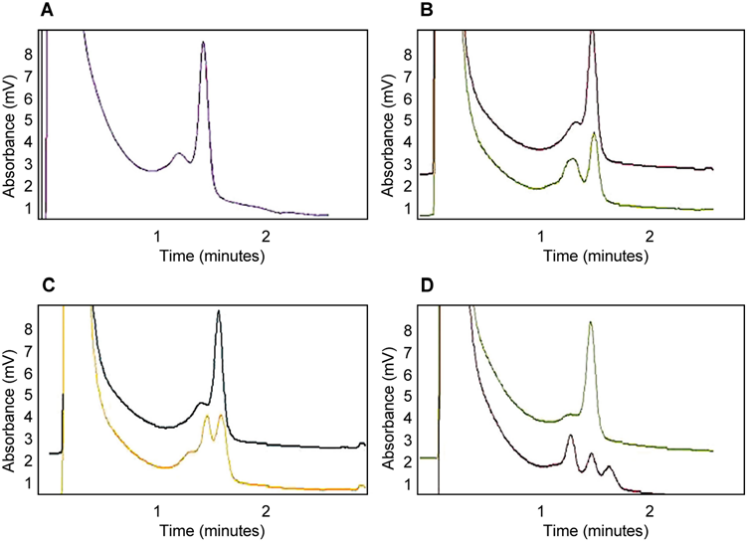
Mutation detection in the T4_promoter fragment. Three polymorphisms were detected by dHPLC in the T4_promoter fragment and were verified by DNA sequencing. The four dHPLC graphs correspond to the wild-type sequence (A), SNPs T-441C (B), SNP A-271G (C) and SNP G-259C (D). The wild-type pattern is visible in all dHPLC graphs as a 1 peak trace. SNP T-441C is identified by an unequal height 2 peak trace (B), SNP A-271G is identified by an equal height 2 peak trace (C) and SNP G-259C is identified by a 3 peak trace (D). The positions of the sequence variants in the T4_promoter fragment identified by dHPLC were determined by DNA sequencing. All SNPs were present in the heterozygous state.

**Figure 3:**
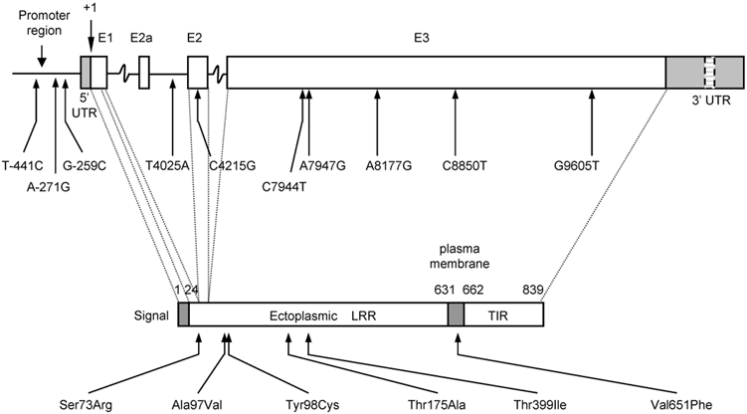
The positions of the *TLR4* polymorphisms in the genomic sequence and the polypeptide sequence. Three polymorphisms were identified in the upstream region (T-441C, A-271G, G-259C), one in intron 2 (T4025A), one in exon 2 (C4215G), and five in exon 3 (C7944T, A7947G, A8177G, C8850T, G9605T). E1, E2, E3, E2a, represent exons 1, 2, 3 and the alternative exon 2. Transcriptional site indicated as +1. 6 exonic polymorphisms cause a change in amino acid residue, with 5 in the ectoplasmic domain and 1 in the plasma membrane domain. LRR denotes; leucine rich repeat. TIR denotes; Toll/IL-1R domain.

For six fragments (T4_E1, T4_E2.1, T4_E3.2, T4_E3.4, T4_E3.5 and T4_E3.7; see figure 1), no trace differing from the wild-type trace was identified in 186 samples. For the remaining five fragments (T4promoter, T4E2.2, T4E3.1, T4E3.3 and T4E3.6; see figure 1) a total of 11 trace patterns differing from the wild-type trace pattern were found in the TLR4 gene; 3 in the promoter fragment, 2 in the exon 2 fragment and 6 in the exon 3 fragment. DNA sequencing confirmed 10 polymorphisms as compared to the reference sequence (NCBI accession number AF177765) and the positions of each sequence change was based on the translational start (the A of ATG being +1) of this TLR4 sequence. Figure 2 shows an example of mutation detection in the T4promoter fragment that displayed four different dHPLC patterns. One dHPLC pattern corresponded to the reference sequence and the other patterns represented 3 polymorphisms, T-441C, A-271G and G-259C. The positions of the polymorphisms in relation to the genomic sequence and the polypeptide sequence are represented in figure 3. Three polymorphisms were identified in the upstream region, one was intronic, and six polymorphisms were identified in exon 3 (figure 3a). All six exonic polymorphisms cause a change in amino acid residue; five are in the ectoplasmic domain and one is in the plasma membrane domain of the TLR4 protein (figure 3b). Seven out of 10 polymorphisms identified in the Vietnamese population are novel, with C8850T (Thr399Ile) being previously reported in Caucasian and other populations (reviewed by [23]). The previously reported A896G (Asp299Gly) was absent in the complete sample set of 744 typhoid cases and controls.

### TLR4 polymorphisms and typhoid fever

dHPLC detects polymorphisms that are present in individuals in the heterozygous state. An additional step of mixing all samples with a reference wild-type sample is necessary to detect polymorphisms in the homozygous state by dHPLC. However, in this study the frequency of each polymorphism, in the heterozygous state in our population (372 cases and 372 controls), was very low (<5%) and as such we determined that it was unnecessary to screen for individuals with homozygous polymorphisms. We calculated that individuals with homozygous polymorphisms would be at an extremely low frequency and it would be highly unlikely they would be detected in our samples size (using sample size calculations; data not shown). The genotype frequencies for all 10 polymorphisms calculated from the detection of mutations in the heterozygous state showed no significant difference from the HWE values expected. Therefore we can assume with confidence that no homozygous polymorphisms were present in the case or controls groups for the 10 polymorphisms genotyped.

Table 3 shows the genotypic and allelic comparisons between typhoid cases and controls.

**Table 3:** Genotypic and allelic comparisons between Typhoid cases and controls.

Mutation	Typhoid case				Control				Allelic comparison			Genotypic comparison 12/11			HWE
	Allele		Genotype		Allele		Genotype		Odd ratio	CI 95%	P	Odd ratio	CI 95%	P	
	Minor (2)	Major (1)	12	11	Minor (2)	Major (1)	12	11							
**G –259 C** rs41391946	18	810	18	396	14	702	14	344	1.11	0.5–2.4	0.76	1.1	0.52–2.46	0.76	1
**A –271 G**	4	824	4	410	0	716	0	358	_	_	0.08	_	_	0.08	1
**T –441 C**	4	824	4	410	5	715	5	355	0.69	0.13–3.23	0.58	0.69	0.14–3.25	0.58	1
**T 4025 A**	**21**	**643**	**21**	**311**	**12**	**704**	**12**	**346**	**1.9**	**0.89–4.3**	**0.07**	**1.9**	**0.89–4.41**	**0.06**	1
**C 4215 G**	**6**	**652**	**6**	**323**	**1**	**725**	**1**	**362**	**6.67**	**0.8–307**	**0.04**	**6.7**	**0.8–310**	**0.04**	1
**C 7944 T**	3	687	3	342	2	654	2	326	1.42	0.16–17.1	0.69	1.4	0.16–17	0.69	1
**A 7947 G**	2	688	2	343	0	656	0	328	_	_	0.26	_	_	0.26	1
**A 8177 G** rs16906079	1	689	1	344	0	656	0	328	_	_	0.51	_	_	0.5	1
**C 8850 T** rs4986791	1	657	1	328	0	568	0	284	_	_	0.54	_	_	0.5	1
**G 9605 T**	2	600	2	299	0	630	0	315	_	_	0.24	_	_	0.24	1
**ND** 1	1	601	1	300	0	630	0	315	_	_	0.49	_	_	0.48	1

1 ND = not done.

A total of 97 heterozygous polymorphic variants were found in TLR4 in the Vietnamese population. Sixty-three heterozygous polymorphic variants were found in the typhoid fever cases and thirty-four in the cord-blood controls. The allele frequencies of all polymorphisms identified in the Vietnamese population were low at <3.2%. The two most common polymorphisms are T4025A with a minor allele frequency of 1.68% (12/716) in controls and 3.16% (21/664) in typhoid cases, and G-259C with a minor allele frequency of 1.95% (14/716) in controls and 2.17% (18/828) in typhoid cases (table 3).

The allelic and genotypic frequencies of C4215G (Ser73Arg) were significantly higher in typhoid fever cases compared to controls (allelic; OR = 6.67, 95%CI = 0.8–307, *P* = 0.04, genotypic; OR = 6.7, 95%CI = 0.8–310, *P* = 0.04; table 3). The odds ratio shows a potentially large effect of this SNP on typhoid susceptibility, however due to the low frequency of the polymorphism and the sample size used in this study, it was of borderline significance. This is not surprising as the power of this sample set (cases N = 372, controls N = 372) to detect an association with C4215G (allele frequency of 0.51, significance of *P* = 0.05 and effect size of OR = 3) is 55.6%. When the allele frequency is as low as 0.51, there is only 9.5% power to detect a modest effect (significance of *P* = 0.05 and effect size of OR = 1.5). The *P* value presented above for C4215G (Ser73Arg) was also not corrected for multiple comparisons. When using Bonferroni correction this association no longer reaches significance (*P*>0.05). In addition, the allelic and genotypic frequencies of T4025A (intronic) showed borderline significance when comparing frequencies in typhoid fever cases and controls (allelic; OR = 1.9, 95%CI = 0.89–4.3, *P* = 0.07, genotypic; OR = 1.9, 95%CI = 0.89–4.41, *P* = 0.06; table 3).

Six non-synonymous SNPs were found in the population, two were present in both case and control groups (C4215G and C7944T) while four were only seen in typhoid cases (A7947G, A8177G, C8850T and G9605T). Five out of six of these non-synonymous SNPs are found in the ectoplasmic domain (leucine rich region) of TLR4 (figure 3b).

## Discussion

Naturally occurring genetic variations in the genes that control innate immunity may play an important role in human susceptibility to a variety of diseases that require an adequate and appropriate immune response. According to Lazarus *et al* (2002) [24], one main consideration to support the above hypothesis is that innate immunity genes are critical for both triggering and sustaining inflammatory responses, and in providing cues necessary to program an adaptive, antigen-specific response. *TLR4* is one innate immunity gene which functions by triggering a signaling cascade inside cells to generate an innate inflammatory response as well as contributing to the development of adaptive immunity [9], [10], [25], [26]. Common variants of *TLR4* that change the function of the protein in the immune system have been previously reported [11]. This has lead to the hypothesis that other genetic variations of *TLR4* may change the function of the protein and alter the efficiency of the immune response to an infectious disease.

Sequence analysis of *TLR4* in various species has revealed that it is highly polymorphic [20], [27]. Smirnova *et al* (2003) [19] reported 13 *TLR4* polymorphisms in a Caucasian population and another different 13 *TLR4* variants were identified in a Dutch population [15]. A common functional *TLR4* mutation Asp299Gly described by Arbour *et al* [11] was not present in the Vietnamese population. Notably, out of the ten mutations identified in the Vietnamese population seven are novel mutations.

Besides the two reported common polymorphisms (Asp299Gly and Thr399Ile), most polymorphisms within *TLR4* occur at low frequencies in populations [15], [19], [20]. Likewise, all mutations detected in our study are low in frequency (<5%). Therefore it is difficult to establish their role in genetic susceptibility to infectious disease. However these mutations may exist at higher frequencies in different ethnic populations and could be useful as candidate SNPs for genetic association studies in other populations. There is little doubt that common host genetic variation present at higher frequencies in the population (minor allele frequency of >5%) can influence the frequency and course of infectious diseases. It is also possible that low frequency SNPs (0.05–5%) may contribute to human disease susceptibility, although this can be difficult to verify, but recent theoretical modeling provides evidence to support this hypothesis [28]–[30]. There is a study which supports the notion that rare as well as common variants of *TLR4* may be associated with infectious disease susceptibility [19]. Within a population with meningococcal disease, a group of rare SNPs with frequencies between 0.003 and 0.0168 were collectively shown to be associated with disease (*P* = 2×10^−6^, odds ratio 27.0), while the frequencies of the common mutations Asp299Gly and Thr399Ile did not differ significantly between cases and controls (*P* = 0.2) [19]. Although this type of analysis may be thought of as controversial, it contributes by highlighting these often ignored rare variants and reports an initial attempt to define the role of rare missense mutations in disease susceptibility.

With rare mutations, it is difficult to assess levels of linkage disequilibrium, as a very small number of individuals carry greater than one mutation. In this sample set only one individual harboured two different mutations and all the remaining mutations appeared in different individuals. Therefore haplotypes generated from these SNPs in this population are uninformative as they would only harbour a mutant allele for one SNP of the haplotype.

In theory, a potential confounding effect of using cord blood controls in genetic association studies is the influence of early childhood mortality on SNP frequencies. It is possible that a gene frequency in the general population could vary with age, however this would require a huge mortality for that specific genotype. The influence of childhood mortality in our study would be small since in Vietnam the <5 year childhood mortality is 17 per 1000 births (1.7%). In HCMC where our cohort is collected this mortality would be lower due to better living conditions and health care, than those experienced nationwide. In addition, a significant percentage of this mortality rate would be due to trauma, congenital and maternal mortality. In practice, previous genetic studies in infectious diseases support the utility of using cord blood controls and include evidence that polymorphism frequencies are comparable between cord blood and adult controls [31]–[33]. Recently the Wellcome Trust Case Control Consortium [34] compared two different population control groups in their large genome wide association study. They reported few significant differences between the two population control groups despite differences in age. Another large genetics consortium, MalariaGEN [35] utilize cord blood controls as population controls in large scale genetic association studies.

Genetic variation can contribute to risk of disease but also to disease outcome. In this study it was not possible to look for enrichment of the coding variants among typhoid patients who exhibited a more severe phenotype ie. longer duration, severity, death. The reasons for this are (1) the frequencies of the mutations are rare, (2) severe complications of typhoid are rare, and are very varied in their phenotype, and (3) death is a rare outcome. In addition the typhoid cases in this study were all recruited as part of a number of clinical intervention trials and as such the treatment of the typhoid case group as a whole was not uniform. The treatment regime for each typhoid case has a significant impact on the duration of disease. If the frequencies of the SNPs were higher in our population we would have had sufficient patient numbers to be able to stratify the typhoid case group by each antibiotic treatment regime, and then analyse the genotypic effect on disease duration.

The TLR family has been described as type I transmembrane pattern recognition receptors (PRR) that possess varying numbers of extracellular N-terminal leucine-rich repeat (LRR) motifs, followed by a cysteine-rich region, a transmembrane domain, and an intracellular Toll/IL-1 R (TIR) motif [36]–[39]. Several lines of evidence argue that TLRs play an important role in innate immunity [40] thus, changes in TLR structure could potentially lead to functional changes. In our study, there were 5 missense mutations identified in the ectoplasmic LRR domain, 1 missense mutation in the transmembrane domain, and 3 polymorphisms in the TLR4 promoter region. That we have identified a number of missense mutations of TLR4 at a very low frequency in the Vietnamese may not be surprising. It is possible that mutations that effect the innate immune function of TLR4, and hence the ability to fight infection, may be under negative selective pressure and therefore not establish as a common variant in the population.

Whether the 3 polymorphisms in the TLR4 promoter region effect TLR4 expression is unknown. Two SNPs (G-259C and T-441C) were equally distributed between typhoid cases and controls, however the frequency of these SNPs (3.9−1.4%) did not allow robust statistical analysis. One low frequency promoter polymorphism was over-represented in cases compared with controls (A-271G). Although the 3 identified promoter polymorphisms were not located in consensus-binding sites [41], we cannot rule out their potential to alter gene regulation.

The extracellular domain of TLRs contains 19–25 tandem copies of the LRR motif and is thought to be directly involved in the recognition of various pathogens [9]. Hyakushima *et al* [42] reported that the extracellular TLR4 region of Glu^24^-Lys^631^ is the functional domain for LPS and MD-2 binding. We identified five low frequency missense mutations (Ser73Arg, Ala97Val, Tyr98Cys, Thr175Ala, Thr399Ile) in the ectoplasmic LRR domain. The amino acid substitutions may alter protein structure and function as the structure and side chains of some of the substituted amino acids differ from wild-type TLR4. One of these, Ser73Arg, showed a slightly higher frequency in typhoid cases than controls, however this association was not robust as it did not remain significant following Bonferroni correction. These LRR region mutations may potentially disturb phosphorylation of TLR4 altering downstream signaling of inflammatory mediator activation, ultimately contributing to disease susceptibility.

The co-segregated mutations Thr399Ile and Asp299Gly, which also lie in the ectoplasmic domain, are significantly associated with a blunted response to inhaled LPS [11] and a variety of diseases [13], [15], [16], [43]. These mutations are common variants with a frequency of >10% in the Caucasian population [23]. In contrast, Thr399Ile occurred in a low frequency in the Vietnamese population, and co-segregation with Asp299Gly was not observed. It is currently unknown whether the two novel missense mutations we identified (Tyr98Cys, Thr175Ala) alter the function of TLR4, as has been shown with Thr399Ile and Asp299Gly.

The transmembrane domain of TLR4 has a critical role in the functional oligomerization of TLR4. A mutation in the hydrophobic region adjacent to the transmembrane domain of TLR4 did not respond to LPS [44]. We identified a low frequency missense mutation, Val651Phe, in the transmembrane domain of TLR4 and the possibility exists that it may alter the function of TLR4 in response to LPS.

In conclusion, the presence of rare missense mutations in the *TLR4* gene, particularly in the extracellular domain, may affect the immune response to disease pathogens. However case control genetic association studies of this size (372 cases and 372 controls) have inadequate power to address the role of rare mutations in disease susceptibility. Short of increasing the sample size to impractical sizes, there is currently no adequate genetic approach to studying the significance of rare mutations (of <1% frequency) in disease susceptibility. The answer may not “simply” lie in increasing the sample size, and new approaches need to be devised.
